# Histological evaluation of five suture materials in the telson ligament of the American horseshoe crab (*Limulus polyphemus*)

**DOI:** 10.7717/peerj.7061

**Published:** 2019-08-01

**Authors:** Ami E. Krasner, Amy Hancock-Ronemus, Larry S. Christian, Emily H. Griffith, Gregory A. Lewbart, Jerry M. Law

**Affiliations:** 1 Hollin Hall Animal Hospital, Alexandria, VA, United States of America; 2 University of Massachusetts at Dartmouth, North Dartmouth, MA, United States of America; 3 Veterinary Services, North Carolina Museum of Natural Sciences, Raleigh, NC, United States of America; 4 Department of Statistics, North Carolina State University, Raleigh, NC, United States of America; 5 Department of Clinical Sciences, North Carolina State University, Raleigh, NC, United States of America; 6 Department of Population Health and Pathobiology, North Carolina State University, Raleigh, NC, United States of America

**Keywords:** *Limulus*, Suture reaction, Telson ligament, Eugenol, Histopathology

## Abstract

An ideal suture material supports healing, minimizes inflammation, and decreases the likelihood of secondary infection. While there are published recommendations for suture materials in some invertebrates, there are no published recommendations for *Limulus polyphemus* or any chelicerate. This study evaluates the histological reaction of horseshoe crabs to five commonly used suture materials: monofilament nylon, silk, poliglecaprone, polydioxanone, and polyglycolic acid. None of the materials were superior with regards to holding nor was there any dehiscence. Nylon evoked the least amount of tissue reaction. This work also provides a histopathological description of the soft membrane at the hinge area between the opisthosoma and telson (telson ligament) and comments on euthanasia with intracardiac eugenol.

## Introduction

Over the last few decades, there has been increasing awareness of invertebrate species in veterinary medicine. Species-specific medical practices allow clinicians to provide the optimal quality of care for each patient. There are limited published research or review articles regarding best practices for caring for the American Horseshoe crab, *Limulus polyphemus*, despite this marine invertebrate’s common use as a laboratory animal and in public aquaria (Smith, Berkson & Barratt, 2002; Leibovitz & Lewbart, 2004; Smith & Berkson, 2005; Gore et al., 2006; Smith, 2012; Tuxbury et al., 2014).

Horseshoe crabs are used as research models due to the ability to extrapolate their anatomy and physiology to other species, their ease of adaptability as laboratory animals, and their unique blood circulatory features. *Limulus*’s large, compound eyes have been successful models in the understanding of mammalian vision (Lui & Passaglia, 2009). The hemolymph of horseshoe crabs is used in the pharmaceutical industry worldwide to reliably ensure the safety of biologicals, pharmaceutical drugs, and medical devices. Horseshoe crab hemolymph is harvested from over 500,000 animals annually to produce *Limulus* amebocyte lysate (LAL), a substance that detects harmful levels of endotoxin in human and veterinary medical products (Grant, 2001; Walls & Berkson, 2003; Novitsky, 2001; Novitsky, 2009; Anderson, Watson III & Chabot, 2013). LAL has other important applications in food safety, disease diagnosis in the clinical laboratory, in ecological monitoring of environmental systems, and in controlling endotoxin in the equipment and procedures to produce pharmaceuticals (Novitsky, 2001). Chitin from the horseshoe crab’s exoskeleton is considered to have healing properties and has been used as an absorbable suture material and for wound dressings for burn victims (Tanacredi, 2001; Smith & Berkson, 2005).

Although horseshoe crabs have persisted for more than 200 million years, the conservation status of free ranging *L. polyphemus* has been evaluated over the last two decades to help ensure this keystone species’ continued survival (Eldredge, 2001; Anderson, Watson III & Chabot, 2013; Smith et al., 2017). The latest assessment suggests that *L. polyphemus* are vulnerable to local extinction based on subregional differences in environmental conditions, threats, and management (Smith et al., 2017). The harvest process for LAL collection may be contributing to local population decline through animal mortality and from decreased female fitness in those returned to the wild during the spawning season (Hurton, Berkson & Smith, 2009; Anderson, Watson III & Chabot, 2013). Currently, the largest harvests of *L*. *polyphemus* are in the American eel (*Anguilla rostrate*), conch (*Busycon* spp.), and whelk commercial fishing industries where they are the most effective bait source (Eagle, 2001; ASMFC, 2009; Anderson, Watson III & Chabot, 2013; Smith et al., 2017). Overharvesting of horseshoe crabs in the fishing industry may also have deleterious effects on migrating shorebirds who rely on horseshoe crab eggs to help fuel their voyage (Eagle, 2001). Changing shoreline dynamics with human development has decreased habitat availability for horseshoe crab breeding grounds and can limit population growth (Hata & Berkson, 2003; Smith et al., 2017). Other factors including climate change, water quality and pollution events, and bycatch are also considered threats to *L. polyphemus* population status (Smith et al., 2017).

Conservation efforts may help protect horseshoe crabs from population decline. There are stricter, although likely inadequate (Smith et al., 2017), regulations on harvesting by the bait fishery and biomedical bleeding industries (ASMFC, 2006; ASMFC, 2010b), considerations for alternative baits and more efficient use of baits, and efforts to implement a new endotoxin test to replace or supplement the LAL test or to make the LAL test more sustainable (Krisfalusi-Gannon et al., 2018). While horseshoe crab populations have stabilized in the Delaware Bay region and increased in abundance in parts of the Southeast, the Northeast region continues to see a decrease in abundance and the stock status is currently undergoing a benchmark assessment (ASMFC, 2015; ASMFC, 2017; Smith et al., 2017). The International Union for Conservation of Nature (IUCN) predicts a 30% decline over the next 40 years (Smith et al., 2017; Krisfalusi-Gannon et al., 2018).

Free-ranging and captive horseshoe crabs can be affected by infectious and non-infectious diseases (Leibovitz & Lewbart, 2004; Smith & Berkson, 2005; Nolan & Smith, 2009). Infectious etiologies causing health problems include algae, fungi, colonial and filamentous cyanobacteria, Gram-negative bacteria, and parasites. Commonly seen green algal infections can affect the carapace and accessory structures including the dorsal arthrodial membrane (over the heart) and telson ligament (the membrane at the base of the telson connecting the opisthosoma to the telson) (Smith & Berkson, 2005; Braverman, Leibovitz & Lewbart, 2012). A significant non-infectious disease of captive horseshoe crabs is panhypoproteinemia, but traumatic injuries, water quality problems, and molting problems are also seen. Traumatic injuries sustained during collection, transport, or overcrowding in captivity can cause puncture wounds, crushing of the exoskeleton, and fractures of the carapace (Smith & Berkson, 2005).

Wound repair and immune defense in the horseshoe crab have been well documented as the migration of granular hemocytes (amebocytes) from the hemolymph to the area of trauma or infection and subsequent clot formation (Bursey, 1977; Clare et al., 1990; Iwanaga, 2002; Iwanaga & Lee, 2005). Horseshoe crabs have innate immune systems where hemocytes respond to pathogens by the exocytosis of large and small granules that contain antimicrobial and coagulation proteins, forming a clot or “coagulum” (Iwanaga, 2002). Hemocytes are large, round to irregularly shaped cells (7 to 11 µm in diameter) with abundant cytoplasm that contains coarse, bright red secretory granules and a darkly basophilic nucleus (Tuxbury et al., 2014). Once activated, hemocytes can become elongated with the cytoplasmic granules no longer apparent and the nucleus can have a distinct halo (Bursey, 1977). The hemolymph of horseshoe crabs and granules in hemocytes both contain a variety of highly efficient defense molecules (Iwanaga, 2002; Iwanaga & Lee, 2005). The defense systems include hemolymph coagulation and coagulum formation, melanization, complement activation, cell agglutination, antimicrobial action, reactive oxygen species formation, and phagocytic action (Iwanaga, 2002).

Horseshoe crab’s sophisticated defense system has helped ensure species survival for over 200 million years (Iwanaga, 2002). Although the life span of an individual horseshoe crab is estimated at 18–22 years (Smith & Berkson, 2005), longevity for captive horseshoe crabs in public aquaria may be only 2–3 years (Tuxbury et al., 2014). Few facilities may track animals as individuals or monitor mortality rates (Tuxbury et al., 2014). As standards of care for aquatic invertebrate medicine continue to advance, appropriate diagnostic testing and treatments may increase longevity among captive horseshoe crabs. Although harvest for the marine life aquarium trade, scientific collection, and educational use is smaller than the bait fishery and biomedical industries (Smith et al., 2017), veterinary management of captive horseshoe crabs may mitigate the need to replace research or public display animals and could increase the return rate and fitness of the biotechnology population to the wild.

When surgery is indicated, an ideal suture material supports healing by preventing suture dehiscence, minimizing inflammation, and decreasing the likelihood of secondary infection (Fossum, 2007). There are published recommendations for suture materials in other invertebrates (Anderson et al., 2010; Salgado et al., 2014), but there are no published recommendations for *L. polyphemus* or any other chelicerate. While surgical epoxy may sufficiently close a wound in the well-mineralized carapace, sutures may be more appropriate for closure of a wound caused by traumatic injury, biopsy for diagnosis of infectious disease, or surgery affecting less mineralized chitinous or non-chitinous tissues (Nolan & Smith, 2009). Making a surgical window to assess the internal structures may be achieved from less mineralized chitinous areas (Nolan & Smith, 2009). Knowledge of the most appropriate suture materials for horseshoe crabs may also be extrapolated for veterinary use in other chelicerate arthropods. This study evaluates the histological reaction of *L. polyphemus* to five suture materials commonly used in veterinary medicine: monofilament nylon, silk, poliglecaprone, polydioxanone, and polyglycolic acid. This paper also comments on a euthanasia method for horseshoe crabs. Although histological descriptions of many clinically relevant organs have been described (Packard, 1880a; Packard, 1880b; Fahrenbach & Merostomata, 1999; Nolan & Smith, 2009), this work provides a further detailed microscopic description of the telson ligament.

## Materials and Methods

Thirty adult *L. polyphemus* were obtained from Pleasant Bay in Chatham, MA and housed at the Marine Resources Center (MRC) of the Marine Biological Laboratory in Woods Hole, MA during August–September, 2009. Animal collections were approved by the Commonwealth of Massachusetts Division of Marine Fisheries, Scientific Permit #152087. The animals were housed together in flow-through holding tanks with sea water from the Great Harbor, Falmouth, Massachusetts. Water temperature, conductivity, salinity, dissolved oxygen, pH, unionized ammonia, nitrate, and nitrite were measured. The following values were obtained: water temperature, 20.5–21.6 °C; conductivity, 46.81; salinity, 32.9–33 ppt; dissolved oxygen, 11.19; pH, 8.02; unionized ammonia, 0 mg/L; nitrite, 0.002 mg/L; nitrate, 4.1 mg/L. Capelin (*Mallotus villosus*) was fed once weekly. After being acclimated in a 1,500 L fiberglass tank for 45 days, 17 female *L. polyphemus* (weight, 540–800 g; maximum carapace width, 16.5–19 cm) were arbitrarily selected from the group to be used in the study: 14 experimental subjects and 3 control subjects. Sex determination was based on pedipalp morphology. During selection, the criteria for exclusion included showing evidence of injury (i.e., cracked carapace or missing legs) or any observable external epibionts. Prior to suture placement, an identification indicator was placed (either by application of one or two colored rubber bands secured to the middle of the telson or a number assignment marked centrally on the carapace with a Sharpie® marker). There was no IACUC review for this study as NCSU IACUC has never required review of the use of invertebrates in research or teaching and it is not required by the Animal Welfare Act, Public Health Service Policy, or NCSU policies. However, measures were taken to try to reduce pain and stress while optimizing data collection.

### Suture placement

**Figure 1:**
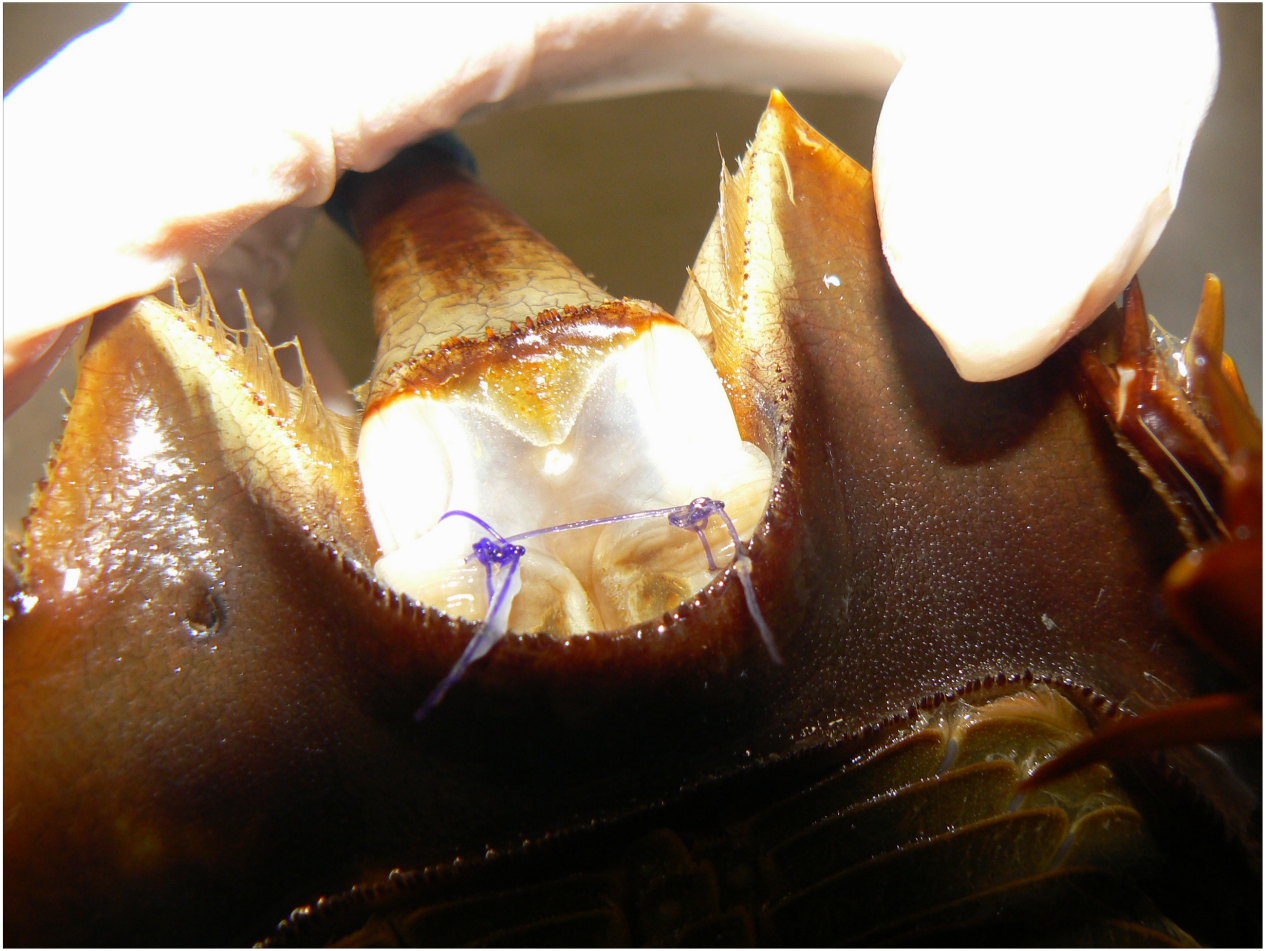
Suture placement (close-up) in the telson membrane of an adult horseshoe crab (*Limulus polyphemus*).

Horseshoe crabs were held in dorsal recumbency and the surgery site (ventral telson ligament) was flushed with sterile saline. The soft cuticle was chosen over the hard cuticle (chitinous exoskeleton) as it would likely have greater knot security and suture placement would more likely be indicated in less or non-chitinous tissues. The ventral telson ligament was chosen specifically based on ease of access to this area. Two simple interrupted sutures were placed, one to the left and one to the right of midline, using one of five suture types in the ventral aspect of the telson ligament (Fig. 1). Sutures were placed to avoid the carapacial margins, which simplified biopsy procedures later. Suture position was rotated among animals so that each suture material was used twice at both sites, *n* = 4 for each suture type. Tested suture materials included monofilament nylon (Polyamide, Grams, Millersville, MD), braided silk (Sofsilk, Ethicon, Summerville, NJ), monofilament poly glycolide-e-caprolactone (Monofyl, Oasis Medical, Mettawa, IL), 3-0 polydioxanone (PDS II, Ethicon, Summerville, NJ), and polyglycolic acid (Webcryl, Webster, Sterling, MA). The three control animals had no sutures placed. The same surgeon (AEK) performed all suture placement procedures. Immediately after suture placement, the horseshoe crabs were transferred to one of two 300 L fiberglass holding tanks and monitored daily for behavior, suture loss, and gross evidence of inflammation or dehiscence at the suture sight.

### Anesthesia/euthanasia protocol

Prior to biopsy collection, local anesthesia with clove oil (2-Methoxy-4-(2-propenyl)phenol, 4-Allyl-2-methoxyphenol, 4-Allylguaiaco, Eugenol, Sigma-Aldrich, St. Louis, MO) was to be administered to all subjects topically with a soaked cotton-tipped applicator to saturate the site (AH Roxanna Smolowitz, pers. comm., 2009). Topical clove oil was applied prior to biopsy for the three control subjects who received no further anesthesia. However, the first biopsy from an experimental subject was associated with uncontrollable hemolymph loss. Thus, systemic injection of clove oil was administered to this subject and prior to biopsy collection in the subsequent thirteen subjects in the experimental group. Two ml of clove oil were injected through the arthrodial membrane of each subject in the experimental group into the cardiac sinus. Higher doses (up to four ml) were administered when needed for immobilization and unresponsiveness of the animal. Time to unresponsiveness to stimuli and suppression of spontaneous movement was noted. Once subjects were unresponsive, biopsy samples were collected. Immediately post-biopsy, experimental group individuals were placed in holding tanks with sea water to facilitate potential spontaneous recovery. Heart rate was monitored via Doppler (Parks Medical Electronics, Aloha, OR) before, immediately after, and a few hours after injection of clove oil in two of the experimental subjects. Doppler readings were taken through the carapace.

### Tissue sample collection

At 6 days following suture placement, half of the experimental group was removed from their holding tanks for biopsy of the suture sites. Biopsies were taken after euthanasia of all animals in the experimental group except for the first experimental subject undergoing sample collection where biopsies were taken before euthanasia. In the three control subjects, a single biopsy was collected from each subject with local anesthesia only. These biopsies were taken from the same sites as suture placement in the experimental group. Excisional biopsies were performed using Metzenbaum scissors; a 3–5 mm diameter tissue sample was obtained by one of two surgeons (AEK or AH) for the control and experimental groups. Control subjects were returned to their holding tanks and the biopsy sites were left to heal by second intention (normal long-term integument healing). After sample collection, biopsies were immediately placed in individual cassettes in 10% neutral buffered formalin. This biopsy procedure was repeated in the remaining half of the experimental group on day 12. Previous suture studies have used similar time frames for gross and histological evaluation, i.e., 6 and 12 days for *Aplysia* (Anderson et al., 2010) and 7 and 14 days for *Xenopus*, *Caretta*, and *Cyprinus* (Hurty et al., 2002; Govett et al., 2004; Tuttle et al., 2006).

The biopsies were processed routinely by the NCSU CVM Histology Laboratory, embedded in paraffin, sectioned at five µm, and stained with hematoxylin and eosin (HE) for evaluation by light microscopy. Multiple sections of each biopsy were evaluated by a single board certified (ACVP) pathologist (JML). There is only one known published description of the normal histology of the telson ligament (Fahrenbach & Merostomata, 1999) and this text is currently out of print. Thus, the pathologist was unblinded to the controls to further characterize the normal histology of the microscopic tissue structure in this area. The pathologist created a five point scale based on our previously used grading scheme for suture reaction studies to describe the microscopic changes in the experimental group (Hurty et al., 2002; Govett et al., 2004; Tuttle et al., 2006; Anderson et al., 2010). Each of the biopsy samples were graded for the amount of inflammation and coagulum formation based on the degree of hemocyte infiltration (Table 1). Once the control subjects were healed, they were returned to the wild.

**Table 1:** 5-point scale for degree of suture reaction of biopsy samples collected 6 and 12 days post suture placement in the telson ligament in *Limulus polyphemus*, *n* = 14.

Score	Descriptions
0	No identifiable microscopic changes or deviations from the non-sutured site
1	Minimal inflammation: one to a few scattered, small aggregates of hemocytes infiltrating the epidermis
2	Mild inflammation: scattered to more numerous activated and granular hemocytes, but loosely arranged and/or spread out
3	Moderate inflammation: more intense aggregates of infiltrating hemocytes
4	Severe inflammation: even more intense, diffuse aggregates of hemocytes with obvious coagulum formation and a very compact cellularity

### Statistical analysis

Summary statistics and frequency counts were calculated for all variables and groupings. Because of the ordinal nature of the histological data, a Kruskal–Wallis nonparametric analysis was performed to compare histological grades (0–4) across suture types for time (day 6 and day 12) separately. *P*-values less than 0.05 were considered to be statistically significant. All analyses were performed in SAS, Version 9.4 (Cary, NC).

## Results

### Anesthesia/euthanasia

Biopsies were collected with local anesthesia for the three control subjects. These subjects had minimal hemolymph loss during biopsy collection. Prior to systemic clove oil injection in the experimental group subjects, heart rate was measured in two of these subjects. Heart rates were 8 bpm and 30 bpm. The 30 bpm subject was very active and the 8 bpm subject was quiet. Immediately after the subjects received two ml of clove oil, no further heartbeats were detected which was confirmed several hours later. Gill movement and slight reflex action in the claws were noted after cessation of cardiac activity was confirmed by Doppler.

Time from systemic injection of clove oil to lack of response to stimuli, cessation of righting reflex, and suppression of spontaneous movement of the claws and gill movement generally ranged from 2–5 min for all subjects in the experimental group. Three subjects had prolonged gill and limb movements and required higher doses of intravascular clove oil; up to 4 mL were administered. None of the experimental subjects spontaneously recovered after systemic clove oil administration.

### Normal histological description of the telson ligament

**Figure 2:**
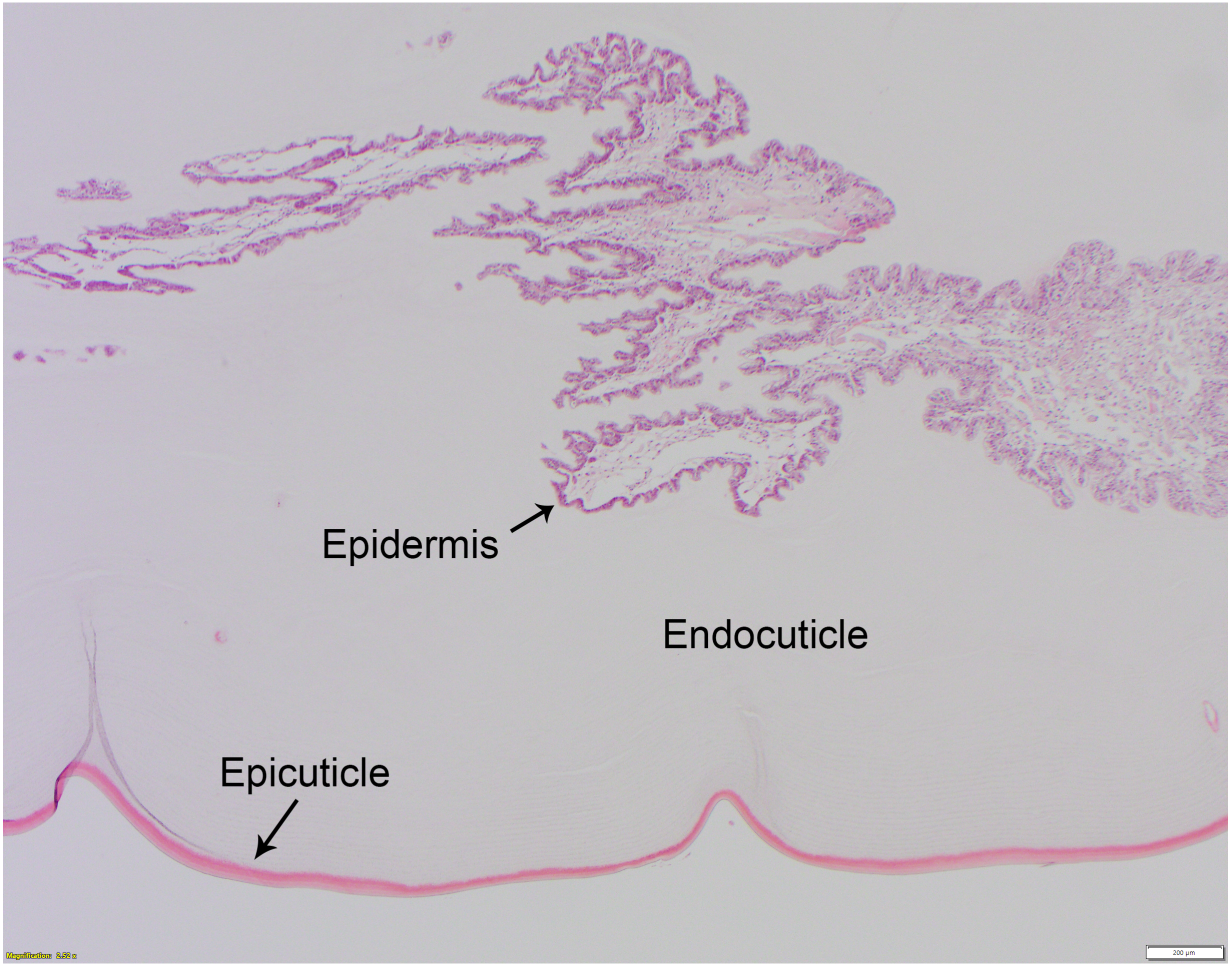
Normal histology of the three-layered telson ligament of the horseshoe crab (*Limulus polyphemus*). The thin (approx. 20–40 μm wide), outer-most epicuticle is brightly eosinophilic, acellular, and has regularly spaced, concave indentations or notches approx. 200–400 μm deep. The endocuticle is the middle layer, is also acellular, and has a pale eosinophilic, finely layered appearance in hematoxylin and eosin-stained (HE) paraffin sections. The epidermis (see also Fig. 3) is basophilic and is composed of tall columnar cells with basally-located nuclei. HE staining; bar = 200 μm.

**Figure 3:**
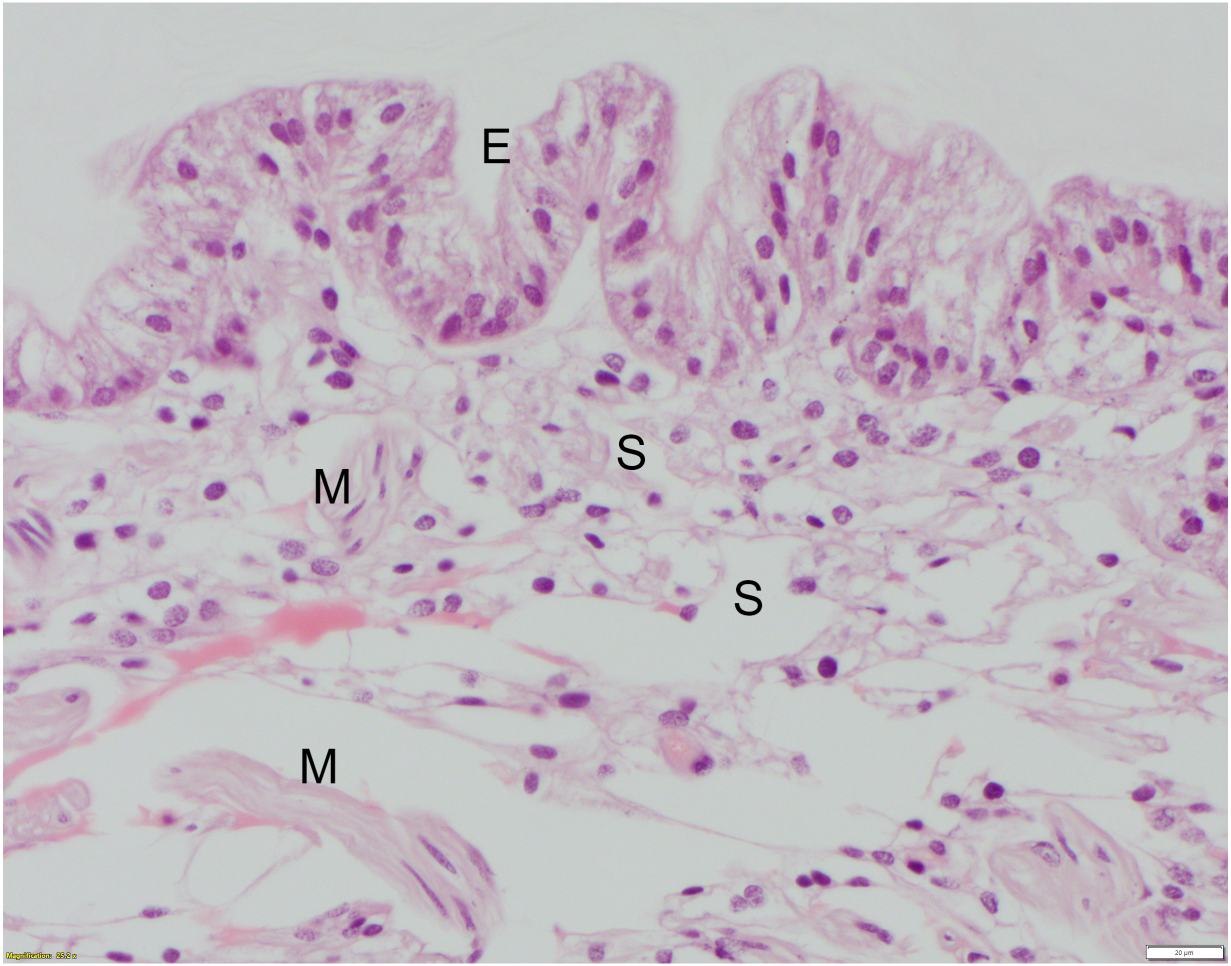
High mag. (40X objective), section of telson ligament that was sutured with monofilament nylon, 6 days post-suturing, scored as grade 0 (no remarkable microscopic changes). The tall columnar cells of the epidermis (E) have clear apical vacuoles, moderately foamy cytoplasm, and basally located nuclei with dense chromatin and no apparent nucleolus. Below the epidermis are the hemolymph sinuses (S) lined by fine fibrocyte septae and interspersed with small clusters of skeletal muscle fibers (M). HE; bar = 20 μm.

The non-chitinous telson ligament has variable thickness and is composed of three distinct layers (Fig. 2). The epicuticle- or outermost layer- is thin (approx. 20–40 µm wide in paraffin sections), brightly eosinophilic, and acellular; it appears to have regularly spaced indentations. The endocuticle- or middle layer- is pale, lightly eosinophilic, acellular, and much thicker than the epicuticle; it has a linear/layered appearance in paraffin sections. The epidermis- or deepest layer- is predominantly basophilic and consists of tall columnar epithelial cells with clear apical vacuoles, moderately foamy cytoplasm, and basally located nuclei with dense chromatin and no apparent nucleolus (Fig. 3). Beneath the epidermis lies a network of hemolymph sinuses lined by a matrix of loosely organized connective tissue septae and interspersed with thin striated muscle fibers (Figs. 2 and 3).

### Suture study

**Figure 4:**
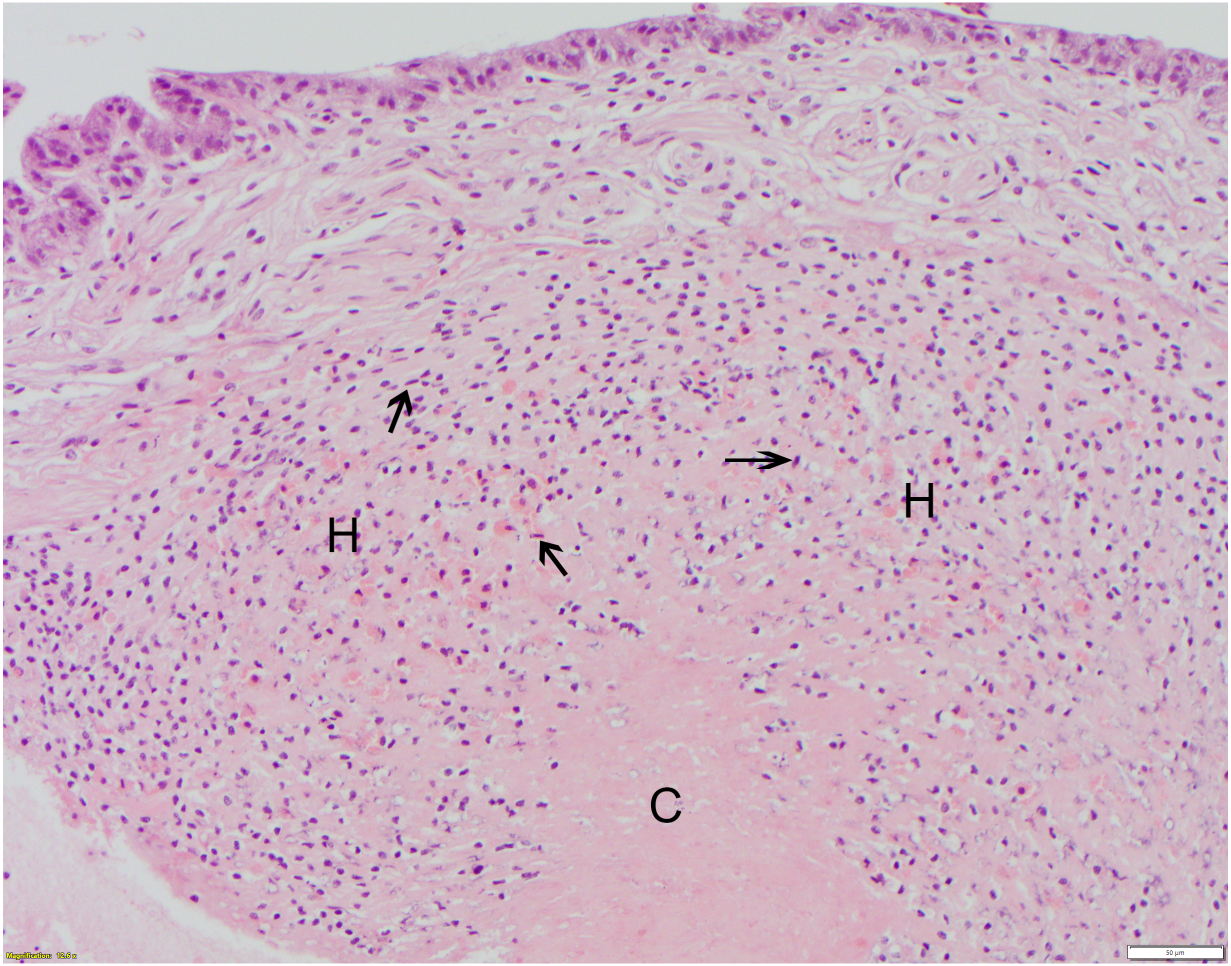
Polyglycolic acid (Webcryl), grade 2 suture reaction, 12 days post-suturing. At bottom center, the healing wound is dominated by a dense, proteinaceous hemolymph coagulum (C) which is paucicellular centrally. This is flanked by moderate infiltrates of granular and agranular (spent) hemocytes (H). Some agranular hemocytes (arrowheads) are elongated with flattened/ovoid nuclei and, in the outer layers, are arranged roughly parallel to each other. The overlying epidermis shows no remarkable abnormalities at this stage of healing. HE; bar = 50 μm.

**Figure 5:**
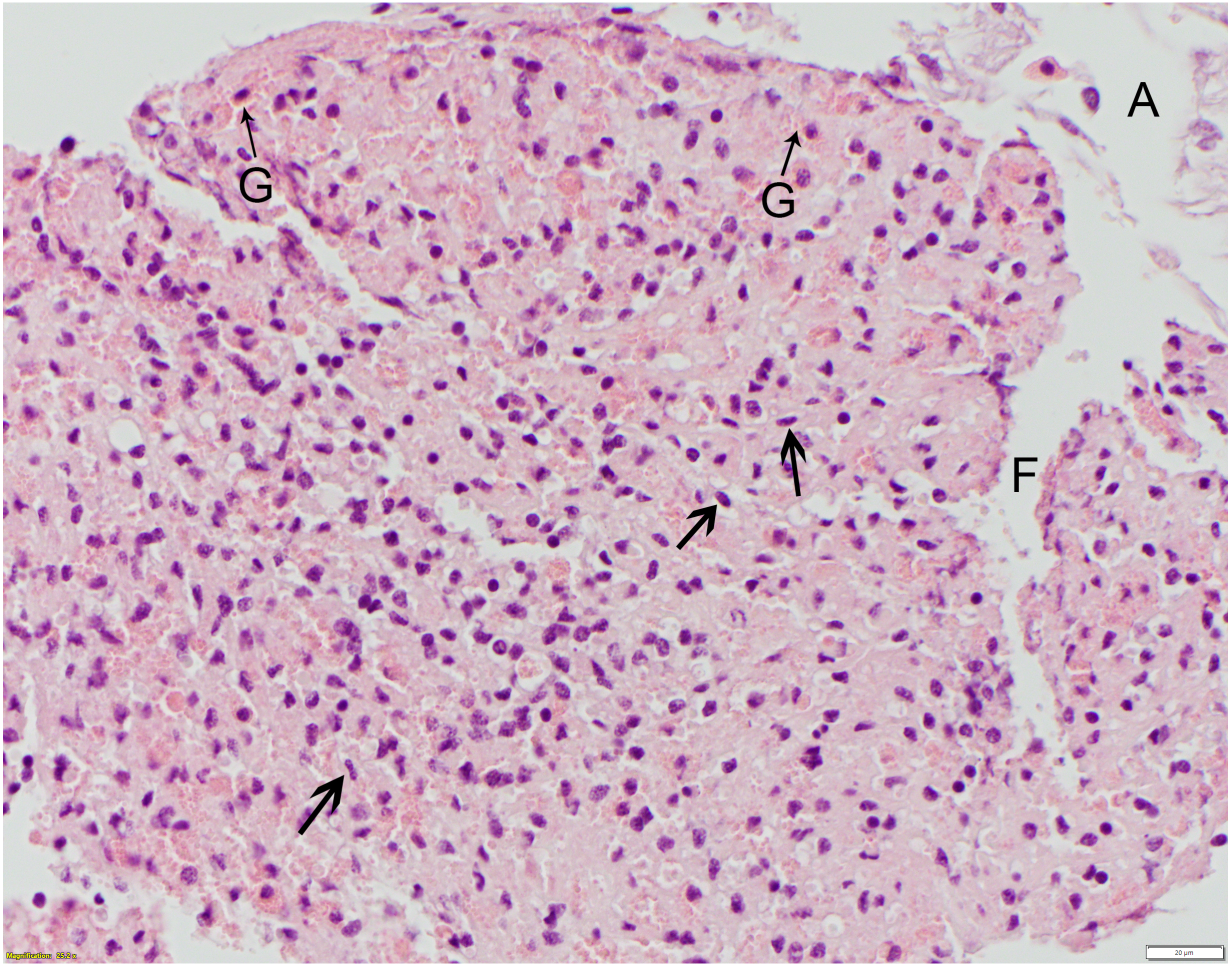
Polydioxanone (PDS II), grade 4 suture reaction, 6 days post-suturing. This high mag. photo shows robust infiltrates of granular (G) and agranular (arrowheads) hemocytes distributed throughout the hemolymph coagulum. Some agranular hemocytes (arrowheads) have angular to stellate to flattened/ovoid nuclei. This photo also illustrates a technical challenge encountered in sectioning some of the telson ligaments, with separation artifact (A) of the endo- and epicuticle layers from the epidermis (not shown) as well as fragmentation (F) of the coagulum. HE; bar = 20 μm.

All suture knots were intact at the time of biopsy. There was no macroscopic evidence of edema or inflammation after suture placement at the suture sites at 6 or 12 days for any suture type. For each suture material, severity scores varied moderately. Reaction scores did not differ significantly between 6 and 12 days or between suture types, but all suture types elicited tissue reactions when compared to the controls (Table 2). Monofilament nylon (Fig. 3) had the lowest mean (1.3) and range (0–2) for histology scores while polyglycolic acid (Fig. 4) had the highest mean (2.3) and range (0–4) for histology scores. No changes were appreciated in the epicuticle or endocuticle, but variable amounts of tissue reaction were seen within the epidermis and hemolymph sinuses. If there were no identifiable microscopic changes, the sample was given a Grade 0 (Fig. 3). Histologic samples representing minimal tissue reaction had one to a few scattered, small aggregates of hemocytes infiltrating the epidermis (Grade 1). Mild reactions (Grade 2, Fig. 4) showed more numerous hemocytes than Grade 1, but these aggregates were loosely arranged and spread out. Figure 4 shows polyglycolic acid at 12 days post-suturing; an area of paucicellular hemolymph coagulum is flanked by loose aggregates of granular and agranular (spent) hemocytes in layers. In the healing wound, flattened agranular hemocytes begin to line up in parallel layers (Bursey, 1977), reminiscent of granulation tissue seen in mammalian wound healing. Moderate tissue reactions (Grade 3) showed more intense aggregates of infiltrating hemocytes and severe tissue reactions (Grade 4) showed even more intense, diffuse aggregates of granular and agranular hemocytes with obvious coagulum formation and a very compact cellularity (Fig. 5). In these cases, numerous hemocytes have lost their granules, often creating “halo cells” (Bursey, 1977) with a perinuclear cytoplasmic clear zone.

**Table 2:** Overall histology scores based on suture type placed in the telson ligament of *Limulus polyphemus*.

Suture type	Histology score	
	Mean	Range
Monofilament nylon	1.3	0–2
Braided silk	1.3	0–4
Poliglecaprone	1.5	1–2
Polydioxanone	2	0–4
Polyglycolic acid	2.3	0–4

## Discussion

Unlike in other invertebrate suture studies (e.g., *Aplysia*, *Lumbricus*), none of the suture types dehisced prior to sample collection (Anderson et al., 2010; Salgado et al., 2014). *Lumbricus* were evaluated at 3 and 6 days post suture application and *Aplysia* were evaluated at 6 and 12 days post suture application. In *Limulus*, while there were variable amounts of tissue reaction in the epidermis, there were no appreciable changes to the epicuticle or endocuticle after suture placement in this study. The stability of the dorsal two layers (particularly the endocuticle) could provide knot security and may account for the lack of observable gross changes identified after suture placement.

The lack of cellularity and hemolymph sinuses in the dorsal two layers may also account for the lack of observable gross changes identified after suture placement. Minimal gross changes were also seen after suture placement in *Aplysia* (Anderson et al., 2010). While inflammation and epidermal changes including necrosis were noted grossly in *Lumbricus*, the tissues lacked the edema that characterizes wound healing in vertebrates (Salgado et al., 2014). Although the histologic response to injury and foreign body insertion has previously been described in horseshoe crabs (Loeb, 1902; Bursey, 1977; Clare et al., 1990), no macroscopic changes to the soft cuticle or hard cuticle were reported. Localized redness as seen in species containing hemoglobin and myoglobin would not be expected. The apparent lack of a grossly visible inflammatory response to injury or foreign materials in the soft cuticle of horseshoe crabs may complicate visual monitoring of healing in this area for clinicians. There are, however, reports of gross changes noted in the hard and soft cuticle secondary to infectious diseases, including tan lesions from *Fusarium solani* infections (Tuxbury et al., 2014) and loss of tissue structure (deformed shells, erosion of the arthrodial membrane, etc.) from green algal, *Chlorophycophytal,* infections (Braverman, Leibovitz & Lewbart, 2012).

Due to the variability in the reaction scores for the epidermis, none of the five suture materials tested were statistically superior for use in *L. polyphemus*. Monofilament nylon elicited the least amount of tissue reaction overall and, thus, may be an acceptable choice as a suture material for horseshoe crabs. Monofilament nylon was recommended as the preferred suture material in the African clawed frog (Tuttle et al., 2006). In other invertebrate species, silk and polyglactin 910 were recommended (Anderson et al., 2010; Salgado et al., 2014). Further studies are recommended to determine if similar variability is repeatable in the telson ligament as well as to determine the recommended suture material in other low or non-chitinous containing tissues. One technical challenge that occurred in our present study is that, despite adequate fixation in 10% neutral buffered formalin and paraffin embedding, some sections had separation of the epi- and endocuticle layers from the epidermis and/or fragmentation of the sub-cuticular tissues, creating artifactual clear spaces (as in Fig. 5). Nonetheless, we were still able to assess and score the suture reactions in the biopsies. In future studies, alternate fixatives and/or embedding in plastic resin such as glycol methacrylate may provide better tissue stability for sectioning and scoring.

Wound healing of the horseshoe crab skeleton differs from that of crustaceans and other invertebrates (Bursey, 1977). There appear to be four phases described by Bursey (1977): (1) lag period, (2) amebocyte (hemocyte) infiltration, (3) hyalinization, and (4) cuticle regeneration. It is likely that a similar process, without the chitinous cuticle formation, occurs in the arthrodial membrane and telson ligament. Hemocyte infiltration and coagulum formation were the most prominent features of wound repair observed in this study. Telson regeneration has also been described (Clare et al., 1990).

Local anesthesia was insufficient for biopsy collection in the experimental subjects due to hemolymph loss. This finding had also been noted in an unpublished pilot study on horseshoe crabs for biopsy collection post suture placement in the telson ligament performed by AH prior to this study. Local anesthesia was sufficient, however, for biopsy collection of the control subjects where no suture had been placed. The increased hemolymph loss in experimental subjects as compared to control subjects may be related to previous migration of hemocytes and a higher hemocyte density in response to suture placement. The infiltration of hemocytes for an extended period of time in response to injury has also been noted in a previous study on horseshoe crabs (Bursey, 1977). This may have implications in appropriate anesthetic procedures and hemostasis during surgery or biopsy collection of horseshoe crabs with diseased or injured tissues.

On average, the *Limulus* heart rate is 32 bpm (Kumar et al., 2015) with a minimum of 12 bpm and maximum of 51 bpm (Redmond, Jorgensen & Bourne, 1982). Prior to systemic clove oil injection, heart rates were measured at 8 bpm and 30 bpm. The 30 bpm subject was very active and the 8 bpm subject was quiet. Previous studies have shown the effects of water temperature, acute hypoxia, and air exposure on heart rates of *Limulus* (Redmond, Jorgensen & Bourne, 1982). The temperature coefficient was within the normal range but bradycardia was seen with acute hypoxia and with air exposure, with a mean reduction of 37.6% in heart rate and a decreased blood pressure noted with air exposure. While baseline heart rates and blood pressures were not measured in this study, the lower than normal heart rate measurements in the two subjects monitored are consistent with the individual subject’s activity level and with exposure to air. Horseshoe crabs’ adaptability to prolonged air exposure likely spans from their annual migration to spawning sites during the mating season. There are no known studies of the effects of air exposure during anesthesia or sedation or with handling stress on the heart rate or blood pressure of horseshoe crabs. Further studies are recommended to determine these effects which can have implications in anesthetic monitoring and safety and other aspects of veterinary care.

Per American Veterinary Medical Association (AVMA) euthanasia guidelines (Leary et al., 2013), clinicians or researchers must choose a method of euthanasia that is rapid, easy to administer, painless, effective, safe, and readily available. Recommendations for euthanasia methods specific to aquatic invertebrates include both physical and chemical methods. Physical methods (i.e., whole body crushing) require pre-anesthesia and many of the chemical methods (i.e., KCl, xylazine, or ketamine injection into the hemolymph sinus) are either controversial or expensive (Leary et al., 2013). Published recommendations for euthanasia specific to horseshoe crabs include pentobarbital injection (390 mg, 1–2 ml/animal) into the cardiac sinus or destruction of the dorsal ganglion located on the dorsal midline between the eyes (Smith, 2012). Success of the dorsal ganglion method is very dependent on proper user technique and time to death after crushing is unknown. Respiratory, cardiac, and cerebral arrest occurs within 30 s after pentobarbital injection (Smith, 2012), but the controlled drug is not always readily available.

Clove oil (Eugenol), a liquid extracted from the leaves of clove trees, has been used as a topical anesthetic by humans for centuries and, more recently, used for invertebrate, amphibian and fish general anesthesia (Gardner, 1997; Treves-Brown, 2000; Goulet, Hélie & Vachon, 2010; Yingdong et al., 2018). Clove oil immersion baths are effective for fish euthanasia (Noga, 2010) and euthanasia of the American giant crab, *Pseudocarcinus gigas*, but the latter may require over 25 min (Gardner, 1997). Dose- and body weight-dependent histological changes were noted in the African clawed frog, *Xenopus laevis*, including renal tubular damage and hepatic necrosis, following clove oil immersion for anesthesia (Goulet, Vachon & Hélie, 2011) and cutaneous necrosis was noted in *Xenopus* after topical application with a Eugenol-soaked gauze (Ross et al., 2006)*.* High concentrations of clove oil are also irritating to human skin (Noga, 2010). As a known hepatotoxin, clove oil may be a safety concern for the handler when used as an immersion versus an injection. There were no identified local histopathology effects of topically administered clove oil noted in the telson ligament in this study, but the degree of systemic absorption and other tissue sites were not evaluated. While there are no known studies of the effects of clove oil on aquatic invertebrate hematology, biochemistry, or histopathology, these effects were studied in the common carp, *Cyprinus carpio* (Velisek et al., 2005). There were no effects on hematologic parameters, but reversible biochemical changes (transient hyperglycemia and increased inorganic phosphates) and histopathology changes (capillary ectasia of the gill filaments) were noted shortly after clove oil immersion although these changes resolved after 24 h. Further research is recommended to evaluate the effects of topical clove oil application, systemic administration of clove oil into the cardiac sinus, and immersion in clove oil baths for sedation, anesthesia and euthanasia on horseshoe crab hemocytes, blood chemistries, biological parameters (i.e., heart rate and blood pressure), and chitin and non-chitin containing tissues. It is also recommended to further evaluate the effectiveness of clove oil for local and systemic anesthesia. The findings in this study suggest that intracardiac injections of clove oil (2–4 ml/kg) can be a humane, economical, accessible, and safe alternative to pentobarbital for euthanasia of horseshoe crabs.

## Conclusions

None of the five suture materials (monofilament nylon, silk, poliglecaprone, polydioxanone, and polyglycolic acid) were superior with regards to holding in the telson ligament and no dehiscence was observed. Nylon caused the least amount of tissue reaction and would be our recommended first choice for use in a chelicerate in need of soft tissue wound repair. Intracardiac Eugenol at a dose of 2–4 mL/kg was determined to be a relatively fast and effective means of horseshoe crab euthanasia.

##  Supplemental Information

10.7717/peerj.7061/supp-1 Supplemental Information 1 The data scoring for the various suture materials from each microscopic slide Each horizontal column represents a glass slide and the pathologist’s interpretation.

10.7717/peerj.7061/supp-2 Supplemental Information 2 The trials on each of the horseshoe crabs Information on the animals, sampling times, and results from the five sutures tested.

10.7717/peerj.7061/supp-3 Supplemental Information 3 Biopsy diagram for processing the telson membrane samples This drawing by the pathologist (Law) describes how he would like the biopsies handles prior to embedding for histopathology.

## Additional Information and Declarations

### Field Study Permissions

The following information was supplied relating to field study approvals (i.e., approving body and any reference numbers): Field experiments were approved by the Commonwealth of Massachusetts Division of Marine Fisheries, Scientific Permit #152087.

### Data Availability

The following information was supplied regarding data availability: All of the raw data related to the suture and histopathology trials are available in the Supplemental File.

## References

[ref-1] Anderson ET, Davis AS, Law JM, Lewbart GA, Christian LS, Harms CA (2010). Gross and histologic evaluation of five suture materials in the skin and subcutaneous tissue of the California sea hare (*Aplysia californica*). Journal of the American Association for Laboratory Animal Science.

[ref-2] Anderson RL, Watson III WH, Chabot CC (2013). Sublethal behavioral and physiological effects of the biomedical bleeding process on the American horseshoe crab, *Limulus polyphemus*. Biological Bulletin.

[ref-3] Atlantic States Marine Fisheries Commission (ASMFC) (2006). Addendum IV to the fishery management plan for horseshoe crab. fishery management report No. 32d of the Atlantic States Marine Fisheries Commission.

[ref-4] Atlantic States Marine Fisheries Commission (ASMFC) (2009). Horseshoe crab stock assessment report.

[ref-5] Atlantic States Marine Fisheries Commission (ASMFC) (2010b). Addendum VI to the fishery management plan for horseshoe crab. Fishery management report No. 32f of the Atlantic States Marine Fisheries Commission.

[ref-6] Atlantic States Marine Fisheries Commission (ASMFC) (2015). 2015 Review of the atlantic states marine fisheries commission fisheries management plan for horseshoe crab (*limulus polyphemus*) 2014 fishing year.

[ref-7] Atlantic States Marine Fisheries Commission (ASMFC) (2017). Horseshoe crab. http://www.asmfc.org/species/horseshoe-crab.

[ref-8] Braverman H, Leibovitz L, Lewbart GA (2012). Green algal infection of American horseshoe crab (*Limulus polyphemus*) exoskeletal structures. Journal of Invertebrate Pathology.

[ref-9] Bursey CR (1977). Histological response to injury in the horseshoe crab, *Limulus polyphemus*. Canadian Journal of Zoology.

[ref-10] Clare AS, Lumb G, Clare PA, Costlow JD (1990). A morphological study of wound repair and telson regeneration in postlarval *Limulus polyphemus*. Invertebrate Reproductive Development.

[ref-11] Eagle J, Tanacredi JT (2001). Issues and approaches in regulation of the horseshoe crab fishery. *Limulus* in the limelight: a species 350 million years in the making and in Peril?.

[ref-12] Eldredge N, Tanacredi JT (2001). Preserving a living fossil. *Limulus* in the limelight: a species 350 million years in the making and in Peril?.

[ref-13] Fahrenbach WH, Merostomata P, Harrison FW, Locke M (1999). Microscopic anatomy of invertebrates.

[ref-14] Fossum TW (2007). Small animal surgery.

[ref-15] Gardner C (1997). Options for immobilization and killing crabs. Journal of Shellfish Research.

[ref-16] Gore SR, Hadfield CA, Clayton LA, Clews A (2006). Challenges of managing horseshoe crabs (*Limulus polyphemus*) in an interactive exhibit.

[ref-17] Goulet F, Hélie P, Vachon P (2010). Eugenol anesthesia in African clawed frogs (*Xenopus laevis*) of different body weights. Journal American Association of Lab Animal Science.

[ref-18] Goulet F, Vachon P, Hélie P (2011). Evaluation of the toxicity of eugenol at anesthetic doses in african clawed frogs (*Xenopus laevis*). Toxicologic Pathology.

[ref-19] Govett PD, Harms CA, Linder KE, Marsh JC, Wyneken J (2004). Effect of four different suture materials on the surgical wound healing of loggerhead sea turtles, *Caretta caretta*. Journal of Herpetological Medicine and Surgery.

[ref-20] Grant D, Tanacredi JT (2001). Living on *limulus*. *Limulus* in the limelight: a species 350 million years in the making and in peril?.

[ref-21] Hata D, Berkson J (2003). Abundance of horseshoe crabs (*Limulus polyphemus*) in the Delaware Bay area. Fisheries Bulletin.

[ref-22] Hurton L, Berkson J, Smith S, Tanacredi JT, Botton ML, Smith DR (2009). The effect of hemolymph extraction volume and handling stress on horseshoe crab mortality. Biology and conservation of horseshoe crabs.

[ref-23] Hurty CA, Brazik DC, Lewbart GA, McHugh Law J, Sakamoto K (2002). Evaluation of the tissue reactions in the skin and body wall of koi (*Cyprinus carpio*) to five suture materials. Veterinary Record.

[ref-24] Iwanaga S (2002). The molecular basis of innate immunity in the horseshoe crab. Current Opinion in Immunology.

[ref-25] Iwanaga S, Lee BL (2005). Recent advances in the innate immunity of invertebrate animals. Journal of Biochemistry and Molecular Biology Research.

[ref-26] Krisfalusi-Gannon J, Ali W, Dellinger K, Robertson L, Brady TE, Goddard MKM, Tinker-Kulberg R, Kepley CL, Dellinger AL (2018). The role of horseshoe crabs in the biomedical industry and recent trends impacting species sustainability. Frontier Marine Science.

[ref-27] Kumar V, Roy S, Sahoo AK, Behera BK, Sharma AP (2015). Horseshoe crab and its medicinal values. International Journal of Current Microbiology and Applied Sciences.

[ref-28] Leary S, Underwood W, Anthony R, Anthony R, Cartner S, Corey D, Grandin T, Greenacre C, Gwaltney-Brant S, McCrackin MA, Meyer R, Miller D, Shearer J, Yanong R (2013). AVMA Guidelines for the euthanasia of animals: 2013 edition.

[ref-29] Leibovitz L, Lewbart GA, Shuster Jr CN, Barlow RB, Brockmann HJ (2004). Diseases and symbionts: vulnerability despite tough shells. The American horseshoe crab.

[ref-30] Loeb L (1902). On the blood lymph cells and inflammatory processes of *limulus*. The Journal of Medical Research.

[ref-31] Lui JS, Passaglia CL (2009). Using the horseshoe crab, *Limulus polyphemus*, in vision research. Journal of Visualized Experiments.

[ref-32] Noga E (2010). Pharmacopoeia. Fish diseases: diagnosis and treatment.

[ref-33] Nolan MW, Smith SA, Tanacredi JT, Botton ML, Smith DR (2009). Clinical evaluation, common diseases, and veterinary care of the horseshoe crab, *limulus polyphemus*. Biology and conservation of horseshoe crabs.

[ref-34] Novitsky TJ, Tanacredi JT (2001). Biomedical products from horseshoe crabs. *Limulus* in the limelight: a species 350 million years in the making and in Peril?.

[ref-35] Novitsky TJ, Tanacredi JT, Botton ML, D Smith (2009). Biomedical applications of Limulus Amebocyte Lysate. Biology and conservation of horseshoe crabs.

[ref-36] Packard AS (1880a). The anatomy histology and embryology of *Limulus polyphemus*. Memoirs of the Boston Society of Natural History.

[ref-37] Packard AS (1880b). Structure of the eye of Limulus. American Naturalist.

[ref-38] Redmond JR, Jorgensen DD, Bourne GB, Bonaventura J, Bonaventura C, Tesh S (1982). Circulatory physiology of Limulus. Physiology and biology of horseshoe crabs.

[ref-39] Ross A, Guenette SA, Helie P, Vachon P (2006). Case of cutaneous necrosis in African clawed frogs Xenopus laevis after the topical application of eugenol. Canadian Veterinary Journal.

[ref-40] Salgado MA, Lewbart GA, Christian LS, Griffith EH, Law JM (2014). Evaluation of five different suture materials in the skin of the earthworm (*Lumbricus terrestris*). SpringerPlus.

[ref-41] Smith DR, Brockmann HJ, Beekey MA, King TL, Millard MJ, Zaldivar-Rae J (2017). Conservation status of the american horseshoe crab, (*Limulus polyphemus*): a regional assessment. Reviews in Fish Biology and Fisheries.

[ref-42] Smith SA, Lewbart GA (2012). Horseshoe crabs. Invertebrate medicine.

[ref-43] Smith SA, Berkson J (2005). Laboratory culture and maintenance of the horseshoe crab (*Limulus polyphemus*). Laboratory Animal.

[ref-44] Smith SA, Berkson JM, Barratt RA (2002). Horseshoe crab (*Limulus polyphemus*) hemolymph, biochemical and immunological parameters. Proceedings of the International Association for Aquatic Animal Medicine.

[ref-45] Tanacredi JT, Tanacredi JT (2001). Horseshoe crabs imperiled?. Limulus in the limelight: a species 350 million years in the making and in Peril?.

[ref-46] Treves-Brown KM (2000). Applied fish pharmacology.

[ref-47] Tuttle AD, Law JM, Harms CA, Lewbart GA, Harvey SB (2006). Evaluation of the gross and histological reactions to five commonly used suture materials in the skin of the African clawed frog (*Xenopus laevis*). Journal of the American Association of Laboratory Animal Science.

[ref-48] Tuxbury KA, Shaw GC, Montali RJ, Clayton LA, Kwiatkowski NP, Dykstra MJ, Mankowski JL (2014). Fusarium solani species complex associated with carapace lesions and branchitis in captive American horseshoe crabs *Limulus polyphemus*. Diseases of Aquatic Organisms.

[ref-49] Velisek J, Svobodova Z, Piackova V, Groch L, Nepejchalova L (2005). Effects of clove oil anesthesia on common carp (*Cyprinus carpio* L). Veterinarni Medicina.

[ref-50] Walls BA, Berkson JM (2003). Effects of blood extraction on horseshoe crabs (*Limulus polyphemus*). Fisheries Bulletin.

[ref-51] Yingdong L, Qiuxin S, Zhibin H, Na S, Xu L, Xiadong L (2018). Anaesthetic effects of eugenol on grass shrimp (*Palaemonetes sinensis*) of different sizes at different concentrations and temperatures. Scientific Reports.

