# Supplementary material for: Histological evaluation of five suture materials in the telson ligament of the American horseshoe crab (Limulus polyphemus)
Source: PeerJ. 2019 Aug 1;7:e7061. doi: 10.7717/peerj.7061 (PMC6679907; doi:10.7717/peerj.7061)
Supplement: Supplemental Information 3 — This drawing by the pathologist (Law) describes how he would like the biopsies handles prior to embedding for histopathology. [file peerj-07-7061-s003.pdf]

School of Veterinary Medicine
NORTH CAROLINA STATE UNIVERSITY

MEMORANDUM

ATTACHED PAPERS

- ____ Please note and return.
- ____ Return with recommendations.
- ____ For your records/information.
- ✓ Speak to me concerning.
- ✓ Please handle.
- ____ For your approval/signature.
- ____ Please reply, sending me a copy.

To Shane

I checked over these, Limulus (HC) samples. I would remove the suture, then bisect the tissue, through the suture hole area, in longitudinal plane:

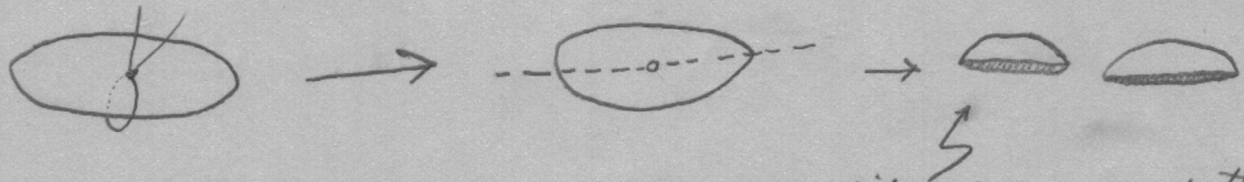

Histo lab can embed the tissue, cut surface down, so we see the cut surface area & sutured area.

Date 9/23/09

Signed: MacLaw
